# Global, regional, national prevalence, mortality, and disability-adjusted life-years of cutaneous squamous cell carcinoma and trend analysis from 1990 to 2021 and prediction to 2045

**DOI:** 10.3389/fonc.2025.1523169

**Published:** 2025-02-06

**Authors:** Chengling Liu, Xingchen Liu, Pengjuan Cao, Xin Li, Haiming Xin, Sailin Zhu

**Affiliations:** ^1^ Center of Burns and Plastic Surgery and Dermatology, The 924th Hospital of Joint Logistics Support Force of the Chinese People's Liberation Army (PLA), Guilin, China; ^2^ Department of Pathology, Changhai Hospital, Naval Medical University, Shanghai, China; ^3^ Department of Endocrinology and Traditional Chinese Medicine, The 924th Hospital of Joint Logistics Support Force of the Chinese People's Liberation Army (PLA), Guilin, China

**Keywords:** cutaneous squamous cell carcinoma, global burden of disease, frontier analysis, bayesian age-period-cohort (BAPC) model, decomposition analysis, inequality analysis

## Abstract

**Background:**

A serious worldwide health concern is cutaneous squamous cell carcinoma (cSCC). For the purpose of creating focused strategies, it is essential to comprehend geographical variations in cSCC prevalence and trends.

**Methods:**

This study utilized data from the 2021 Global Burden of Diseases (GBD) survey to analyze cSCC across 204 countries and territories. We assessed the age-standardized prevalence rate (ASPR), mortality rate (ASMR), disability-adjusted life years (ASDR), and estimated annual percentage changes (EAPCs), with trends stratified by region, country, age, sex, and Sociodemographic Index (SDI). To evaluate disparities in cSCC burden, we combined the SDI with the inequality slope and concentration indices for an international health inequality analysis. Decomposition analysis assessed the effects of population growth, aging, and epidemiological trends on disease burden, while frontier analysis linked cSCC outcomes with socio-demographic development. A Bayesian Age-Period-Cohort (BAPC) model projected future prevalence, mortality, and DALYs, identifying key drivers of cSCC burden.

**Results:**

In 2021, there were 2,275,834 cases of cSCC globally, reflecting a 345% increase since 1990. During this period, the ASPR rose from 14.69 to 26.85 per 100,000, while the ASMR increased slightly from 0.67 to 0.69 per 100,000. Disability-adjusted life years (DALYs) rose from 544,973 to 1,210,874. Among socio-demographic regions, the high SDI region had the highest ASPR, while the middle SDI region exhibited the highest ASMR and ASDR. Decomposition analysis identified population growth and demographic aging as key drivers of the rising ASMR. Countries like Georgia showed significant disparities in frontier analysis, indicating potential for better cSCC management. Health inequality analysis confirmed that the burden was concentrated in nations with higher SDI. By 2045, the global ASPR is projected to reach 64.66, with the ASMR and ASDR expected to decrease to 1.02 and 20.63 per 100,000, respectively.

**Conclusion:**

Over the last three decades, the global burden of cSCC has increased significantly. While mortality rates and DALYs are expected to decline over the next twenty years, the prevalence of cSCC is projected to remain high. This highlights the urgent need to reevaluate preventive efforts aimed at reducing morbidity, particularly in areas with substantial populations over the age of 95.

## 1 Introduction

With increasing incidence rates and severe morbidity and death, cutaneous squamous cell carcinoma (cSCC) has become a major global public health problem (1). Despite its prevalence, cSCC has often been overshadowed by research on melanoma, leaving considerable gaps in our understanding of its global burden, temporal trends, and regional disparities (2). As the second most common form of skin cancer, cSCC accounts for a growing number of healthcare encounters, which places a significant strain on healthcare systems, especially in regions with high ultraviolet (UV) exposure (1, 3). cSCC has been extensively studied at national and regional levels; however, the global burden and socio-demographic determinants remain underexplored. The prevalence of cSCC varies significantly by region, with higher incidence rates in countries like Australia, the United States, and New Zealand, where UV radiation exposure is particularly intense and a large proportion of the population is Caucasian (4, 5). However, these regional studies lack a comprehensive global perspective, and many focus solely on descriptive statistics without delving into the more nuanced aspects of disease burden or temporal trends. Moreover, while previous studies have identified risk factors such as UV exposure, immunosuppression, and older age, the research is often fragmented, lacking the integration necessary to reveal patterns over time and across populations and failing to examine disparities in healthcare access, early detection programs, and treatment availability (6, 7).

Building upon this foundation, our study employs innovative methodologies, including decomposition and frontier analyses, to assess the interplay between socio-demographic development and cSCC burden. The disease burden of cSCC includes not only clinical outcomes but also psychological and economic impacts, placing significant strain on healthcare systems (8). While mortality rates are relatively low, the rising incidence contributes to substantial disability-adjusted life years (DALYs) worldwide. However, many studies overlook the burden in lower- Sociodemographic Index (SDI) regions, and longitudinal data on changing trends remain scarce (9). Inequities in healthcare access and exposure to environmental risk factors further complicate efforts to understand cSCC globally (10).

This study leverages data from the Global Burden of Disease (GBD) framework to address these gaps, offering a robust longitudinal perspective on cSCC trends from 1990 to 2021. Key innovations include the use of frontier analysis to evaluate healthcare performance relative to economic resources, decomposition analysis to disentangle contributions of demographic factors to disease trends, and inequality analysis to explore disparities across regions and socio-economic groups (11, 12). Through these approaches, the study aims to answer critical questions about global cSCC trends, regional variations, and the factors driving temporal changes.

## 2 Methods

### 2.1 Data sources

This cross-sectional study used data from the Global Burden of Disease (GBD) 2021 database, which is available at https://vizhub.healthdata.org/gbd-results/. It was authorized by the 924th Hospital of the Joint Logistics Support Force of the Chinese People’s Liberation Army (PLA) without needing informed permission. The GBD 2021 research used the most recent epidemiological data and improved standardized procedures to thoroughly evaluate health loss across 204 nations and regions (13, 14). Three hundred seventy-one diseases and harms were shown to be major causes of health loss. Cutaneous squamous cell carcinoma (cSCC), characterized within the GBD framework as a non-melanoma skin cancer, classified under ICD(International Classification of Diseases)-10: C44, was studied in individuals aged 15 to 95+ years using data from the Global Health Data Exchange (4).

The GBD 2021 project provided the data used in this investigation, which covered cSCC prevalence, mortality, and Disability adjusted life years (DALYs) from 1990 to 2021. Through a thorough approach that includes systematic literature reviews, hospital records, insurance claims, and national health surveys, the GBD project collects data on global health (15). To produce the most reliable estimates of global health, the GBD employs a strict approach. It ensures measurement comparability across time and geographies by taking illnesses and hazards into consideration. The GBD Results Tool provided data on cSCC mortality, prevalence, and DALYs from 1990 to 2021 (16). The regions comprised the worldwide region as well as five socialdemographic index (SDI) areas. To aid in health research and policy creation, the SDI divides regions into high, high-middle, medium, low-middle, and low categories according to factors including birth rates, income, and education (13). We followed Strengthening the Reporting of Observational Studies in Epidemiology (STROBE) rules and used linear regression to estimated annual percentage change (EAPC).

### 2.2 Statistical analysis

We used published estimates of global, regional, and national prevalence, mortality, and DALYs for the cSCC, disaggregated by sex, location, 5-year age group, and year, based on GBD 2021 data. DALYs integrate years lived with disability and years of life lost to provide a comprehensive evaluation of the total health burden, accounting for both years lost to premature death and years lived with impairment due to a specific health condition (17). In-depth computation methods for age-standardized rates, DALYs, incidence, and prevalence are provided in earlier publications. The GBD 2021 global population age standard per 100,000 people was used as the basis for the direct method’s calculation of age-standardized rates (ASRs) for cSCC. Among these were the age-standard DALY rate (ASDR), prevalence rate (ASPR), and mortality rate (ASMR) (18).

We identified worldwide ASR inflection points from 1990 to 2021 using Joinpoint analysis in order to evaluate EAPC descriptions. Average annual percent change (AAPC) evaluated average annual ASR changes across time, whereas annual percent change (APC) monitored percentage changes within certain sectors. By assessing the statistical significance of ASR trend changes, these techniques supported our findings (19).

Decomposition analysis identified the factors causing increases in DALYs and global cSCC mortality between 1990 and 2021. The decomposition analysis used in our study is based on the Das Gupta method, a robust statistical technique widely employed in epidemiological and demographic research (20). This method facilitates the quantitative disaggregation of changes in age-standardized rates (ASRs) into additive components, allowing for the attribution of observed trends to specific factors such as population growth, demographic aging, and changes in epidemiological rates (21). The method enables policymakers to distinguish between structural drivers (e.g., aging and growth) and modifiable risk factors (e.g., UV exposure or access to healthcare), thereby informing targeted intervention strategies. By disentangling the contributions of each factor, this approach provides a clearer picture of the underlying dynamics influencing cSCC trends across regions (22). We examined DALYs and mortality for five SDI areas as well as the worldwide region. The comprehensive equations and procedures are available in earlier works (23, 24).

The Bayesian Age-Period-Cohort (BAPC) model is a powerful statistical tool used to disentangle the effects of age, period, and cohort on disease trends (25). These three factors are often intertwined, making it challenging to isolate their individual contributions using traditional methods. By leveraging Bayesian statistical techniques, the BAPC model provides a robust framework for understanding past trends and projecting future patterns of disease burden (26). Clarifying the effects of age, period, and cohort on outcomes while emphasizing the uncertainty in these estimates is possible by combining previous views with observed data (27, 28). This model was used to forecast cSCC prevalence and DALY rates through 2045 for people aged 15 to 95+ globally and across five SDI regions.

We utilized a quantitative technique known as “frontier analysis” to ascertain the lowest feasible DALYs ASR linked to development status as determined by SDI in order to evaluate the association between DALY rates of cSCC and the SDI (29). We were able to determine the lowest DALY ASRs that could be achieved using this method, taking into account each nation’s SDI. To calculate the frontier, we employed the free disposable hull technique. In particular, the lowest value at each place was established, and to make sure that every point fell inside these lines, the points inside this border were joined by horizontal and vertical lines (30).

Standardized metrics for quantifying absolute and relative gradient inequalities are the inequality slope index and the concentration index, respectively (30). They measure the disparity in how different nations bear the burden of cSCC. By comparing a nation’s DALY rates to its SDI relative position—which is determined by the population’s midpoint in a cumulative distribution sorted by SDI—the inequality slope index is derived using regression analysis. A weighted regression model is used to investigate heteroscedasticity. In order to match the cumulative percentage of DALYs with the population’s cumulative distribution ordered by SDI, the concentration index is computed by numerically integrating the area under the Lorenz curve (31).

The figures in this study were created using the R software package (version 4.2.3) and jD_GBDR (V2.22, Jingding Medical Technology Co., Ltd.).

## 3 Results

### 3.1 Global burden of Cutaneous squamous cell carcinoma

**Figure 1:**
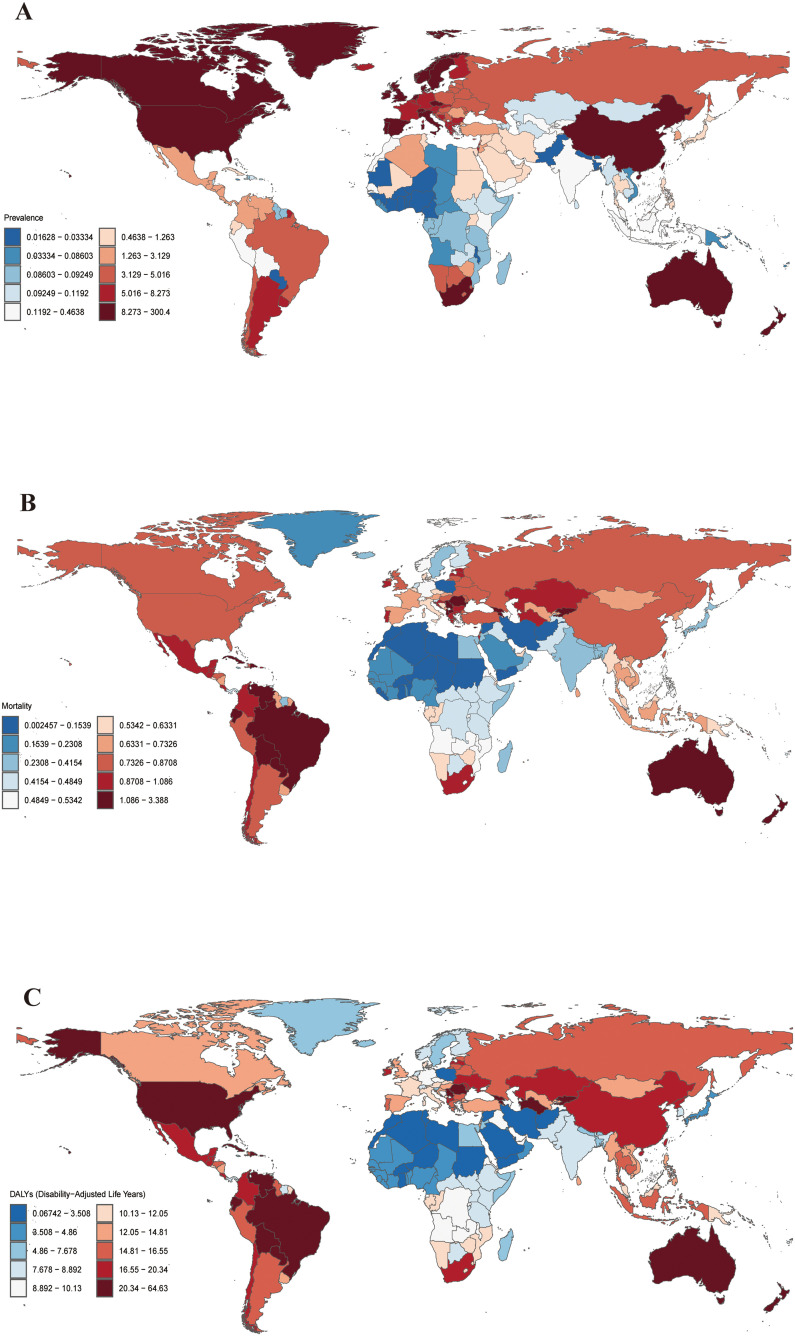
Global ASPR **(A)**, ASMR **(B)** and ASDR **(C)** of cSCC in 2021 (per 100,000 population). ASPR, age-standardized prevalence rate; ASMR, age-standardized mortality rate; ASDR, age-standardized disability-adjusted life year rate; cSCC, cutaneous squamous cell carcinoma.

(**Figures 1A–C**) depicts the worldwide maps of the age-standard DALY rate (ASDR), prevalence rate (ASPR), and mortality rate (ASMR) for cSCC in 2021. ASPR increased the global burden of cSCC between 1990 and 2021. However, ASMR and ASDR increased. Females exhibited lower ASPR, ASMR, and ASDR for cSCC than males in 1990 and 2021 (**Table 1**).

**Table 1:** Age-standardized prevalence rate, DALYs and age-standardized mortality rate of squamous-cell carcinoma between 1990 to 2021 at the global and regional level.

Location	Rate per 100 000 populations (95% UL)						
	1990			2021			1990-2021
	ASPR	ASDR	ASMR	ASPR	ASDR	ASMR	EAPC^a^ of the ASPR
Global	14.69 (11.46-18.34)	14.01 (12.79-15.37)	0.67 (0.60-0.73)	26.85 (22.77-31.77)	14.31 (12.65-15.78)	0.69 (0.59-0.77)	2.29 (1.79-2.78)
Sex							
Female	10.56 (8.37-13.09)	10.61 (9.68-11.75)	0.52 (0.47-0.57)	18.11 (15.42-21.51)	10.80 (9.47-12.02)	0.53 (0.44-0.59)	2.10 (1.57-2.63)
Male	20.73 (16.13-26.12)	18.25 (16.17-20.55)	0.88 (0.78-0.99)	38.18 (32.47-45.10)	18.58 (15.55-20.84)	0.92 (0.77-1.05)	2.30 (1.83-2.79)
SDI							
High	41.17 (32.42-51.10)	15.18 (14.36-16.10)	0.66 (0.61-0.69)	89.98 (76.75-107.24)	14.62 (13.26-16.34)	0.60 (0.53-0.64)	3.13 (2.58-3.68)
High middle	3.65 (2.97-4.52)	16.19 (15.05-18.03)	0.83 (0.75-0.91)	8.71 (6.84-10.77)	15.21 (13.50-17.83)	0.79 (0.69-0.92)	1.13 (0.68-1.58)
Middle SDl	1.53 (1.22-1.89)	15.78 (13.44-17.70)	0.75 (0.64-0.84)	5.63 (4.25-7.17)	16.43 (13.54-18.74)	0.83 (0.68-0.94)	2.32 (1.81-2.82)
Low middle	0.45 (0.35-0.57)	7.91 (6.28-9.82)	0.39 (0.29-0.47)	0.44 (0.33-0.57)	10.54 (8.84-12.24)	0.54 (0.46-0.64)	0.14 (0.06-0.22)
Low SDI	0.17 (0.13-0.23)	5.19 (2.96-6.93)	0.25 (0.14-0.35)	0.18 (0.13-0.24)	7.00 (3.60-9.43)	0.36 (0.19-0.48)	0.10 (0.07-0.12)
Regions							
Andean Latin America	0.62 (0.46-0.81)	11.69 (9.55-14.51)	0.62 (0.50-0.75)	0.35 (0.26-0.48)	20.47 (15.74-24.57)	1.13 (0.87-1.33)	-2.19 (-2.53–1.85)
Australasia	92.63 (72.17-114.19)	31.80 (29.73-34.39)	1.40 (1.29-1.49)	76.67 (59.57-97.05)	31.68 (28.78-34.20)	1.64 (1.44-1.77)	-1.06 (-1.24–0.87)
Caribbean	0.79 (0.65-0.95)	18.05 (16.95-20.55)	0.96 (0.89-1.09)	0.46 (0.35-0.58)	26.25 (22.67-29.45)	1.39 (1.21-1.56)	-2.02 (-2.16–1.89)
Central Asia	0.13 (0.08-0.18)	9.10 (7.43-10.56)	0.46 (0.35-0.54)	0.12 (0.08-0.17)	20.81 (18.62-23.12)	1.12 (0.99-1.23)	-0.27 (-0.30–0.24)
Central Europe	4.42 (3.58-5.47)	27.05 (25.66-28.16)	1.59 (1.48-1.67)	4.54 (3.51-5.70)	11.77 (10.72-12.95)	0.67 (0.60-0.74)	0.26 (0.13-0.39)
Central Latin America	2.26 (1.70-2.90)	26.40 (25.38-27.14)	1.32 (1.25-1.37)	2.07 (1.55-2.69)	18.40 (16.30-20.76)	0.98 (0.87-1.10)	-0.31 (-0.34–0.27)
Central Sub-Saharan Africa	0.09 (0.06-0.13)	6.59 (2.44-9.91)	0.33 (0.12-0.51)	0.09 (0.06-0.12)	9.39 (2.83-14.69)	0.48 (0.15-0.76)	-0.27 (-0.28–0.25)
East Asia	1.55 (1.23-1.90)	16.26 (13.56-20.06)	0.75 (0.63-0.92)	12.15 (9.30-15.24)	17.77 (14.25-21.95)	0.86 (0.67-1.06)	4.22 (3.42-5.03)
Eastern Europe	3.27 (2.55-4.14)	16.05 (15.11-16.74)	0.74 (0.69-0.77)	3.74 (2.84-4.79)	15.73 (14.39-17.09)	0.77 (0.70-0.83)	0.48 (0.43-0.54)
Eastern Sub-Saharan Africa	0.18 (0.13-0.24)	6.34 (2.15-9.63)	0.32 (0.11-0.49)	0.18 (0.13-0.24)	8.58 (2.44-13.25)	0.45 (0.13-0.70)	-0.05 (-0.10–0.01)
High-income Asia Pacific	0.82 (0.62-1.05)	7.28 (6.29-7.78)	0.39 (0.34-0.42)	1.17 (0.87-1.51)	5.26 (4.68-6.47)	0.29 (0.25-0.35)	1.04 (0.96-1.12)
High-income North America	111.30 (86.13-139.04)	23.02 (21.31-25.27)	0.78 (0.73-0.81)	268.76 (231.42-322.06)	26.70 (22.90-31.53)	0.81 (0.73-0.86)	3.58 (2.93-4.24)
North Africa and Middle East	1.10 (0.87-1.38)	4.53 (3.52-6.31)	0.25 (0.19-0.35)	1.04 (0.80-1.31)	4.90 (4.06-6.36)	0.28 (0.23-0.37)	-0.35 (-0.69–0.01)
Oceania	0.04 (0.02-0.07)	10.53 (7.19-14.48)	0.57 (0.39-0.76)	0.04 (0.02-0.07)	12.34 (8.16-17.15)	0.63 (0.42-0.84)	-0.11 (-0.20–0.02)
South Asia	0.20 (0.14-0.28)	6.78 (4.75-9.17)	0.34 (0.22-0.45)	0.21 (0.15-0.29)	7.90 (6.44-10.49)	0.41 (0.33-0.54)	0.15 (0.11-0.20)
Southeast Asia	0.32 (0.26-0.40)	13.15 (10.15-16.01)	0.58 (0.46-0.70)	0.30 (0.23-0.38)	14.40 (11.41-17.20)	0.65 (0.54-0.80)	-0.13 (-0.36-0.10)
Southern Latin America	5.42 (4.31-6.75)	14.68 (13.84-15.59)	0.75 (0.69-0.80)	5.35 (4.07-6.90)	16.05 (14.96-17.08)	0.88 (0.79-0.94)	-0.10 (-0.16–0.04)
Southern Sub-Saharan Africa	7.47 (5.81-9.53)	10.79 (8.19-16.10)	0.55 (0.41-0.81)	10.47 (8.09-13.50)	16.74 (12.90-19.50)	0.84 (0.65-0.96)	0.28 (-0.30-0.87)
Tropical Latin America	3.56 (2.91-4.30)	20.33 (19.41-21.11)	0.96 (0.89-1.01)	3.39 (2.71-4.15)	25.29 (23.38-26.58)	1.31 (1.16-1.41)	0.75 (0.53-0.97)
Western Europe	7.24 (6.01-8.77)	12.30 (11.70-12.68)	0.65 (0.59-0.68)	8.31 (6.37-10.58)	11.11 (10.15-11.71)	0.64 (0.56-0.68)	0.48 (0.36-0.60)
Western Sub-Saharan Africa	0.07 (0.05-0.11)	2.09 (1.11-2.70)	0.09 (0.05-0.11)	0.07 (0.05-0.11)	3.71 (1.18-5.37)	0.15 (0.05-0.21)	0.09 (0.05-0.12)

EAPC, estimated annual percentage change: SDl, Sociodemographic Index; Ul, uncertainty interval; DALY, disability-adjusted life-years.

a EAPC is expressed as 95% Cls.

The prevalence of cSCC increased by 345% from 1990 to 2021, rising from 510,851.30 cases (95% UI: 398,768.11–637,037.00) to 2,275,834.64 cases (95% UI: 1,924,479.29–2,705,755.41). The ASPR similarly saw a 201% increase, going from 14.69 (95% UI: 11.46–18.34) in 1990 to 26.85 (95% UI: 22.77–31.77) in 2021.

In terms of incidence, 465,111.55 cSCC cases (95% UI: 366,707.51–594,410.23) were reported in 1990, which rose to 1,899,907.05 cases (95% UI: 1,688,002.74–2,150,029.57) by 2021—a 308% increase. Correspondingly, the ASIR grew by 67%, rising from 13.38 (95% UI: 10.55–17.26) in 1990 to 22.38 (95% UI: 19.90–25.27) in 2021.

Between 1990 and 2021, the number of cSCC-related mortality cases increased by 151%, from 22,667.02 (95% UI: 20,674.06–24,674.87) to 56,913.23 (95% UI: 48,761.35–63,037.41). The ASMR saw a modest 4% rise, from 0.67 (95% UI: 0.60–0.73) in 1990 to 0.69 (95% UI: 0.59–0.77) in 2021.

DALYs also saw a 122% increase, rising from 544,973.68 (95% UI: 496,193.19–601,306.39) in 1990 to 1,210,874.53 (95% UI: 1,068,480.71–1,334,385.86) in 2021. Meanwhile, the ASDR grew slightly by 2%, from 14.01 (95% UI: 12.79–15.37) in 1990 to 14.31 (95% UI: 12.65–15.78) in 2021.

### 3.2 SDI Regional burden of cSCC

**Figure 2:**
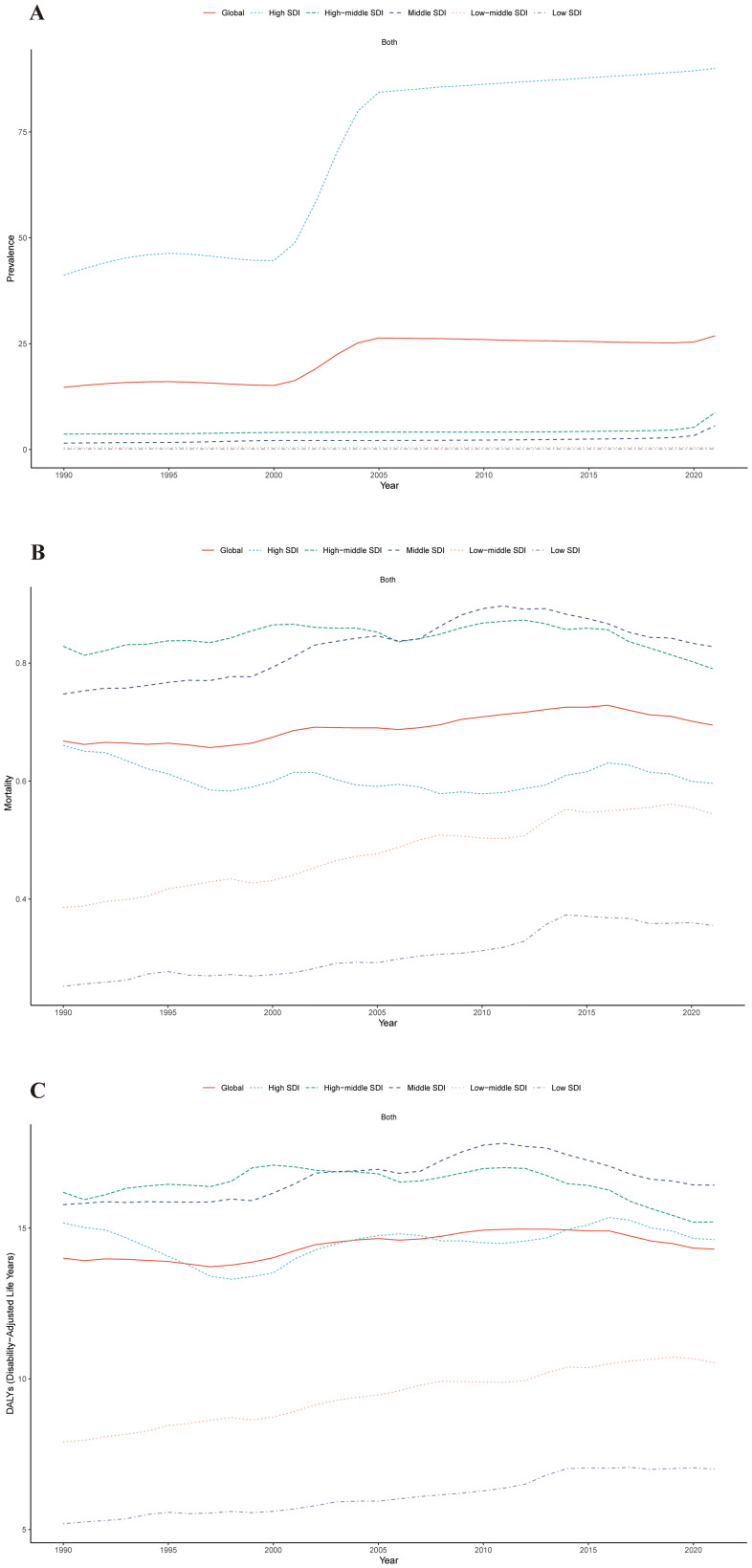
Trends in ASPR **(A)**, ASMR **(B)**, and ASDR **(C)** for cSCC across five Sociodemographic Index (SDI) regions from 1990 to 2021. ASPR, age-standardized prevalence rate; ASMR, age-standardized mortality rate; ASDR, age-standardized disability-adjusted life year rate; cSCC, cutaneous squamous cell carcinoma.

**Figure 3:**
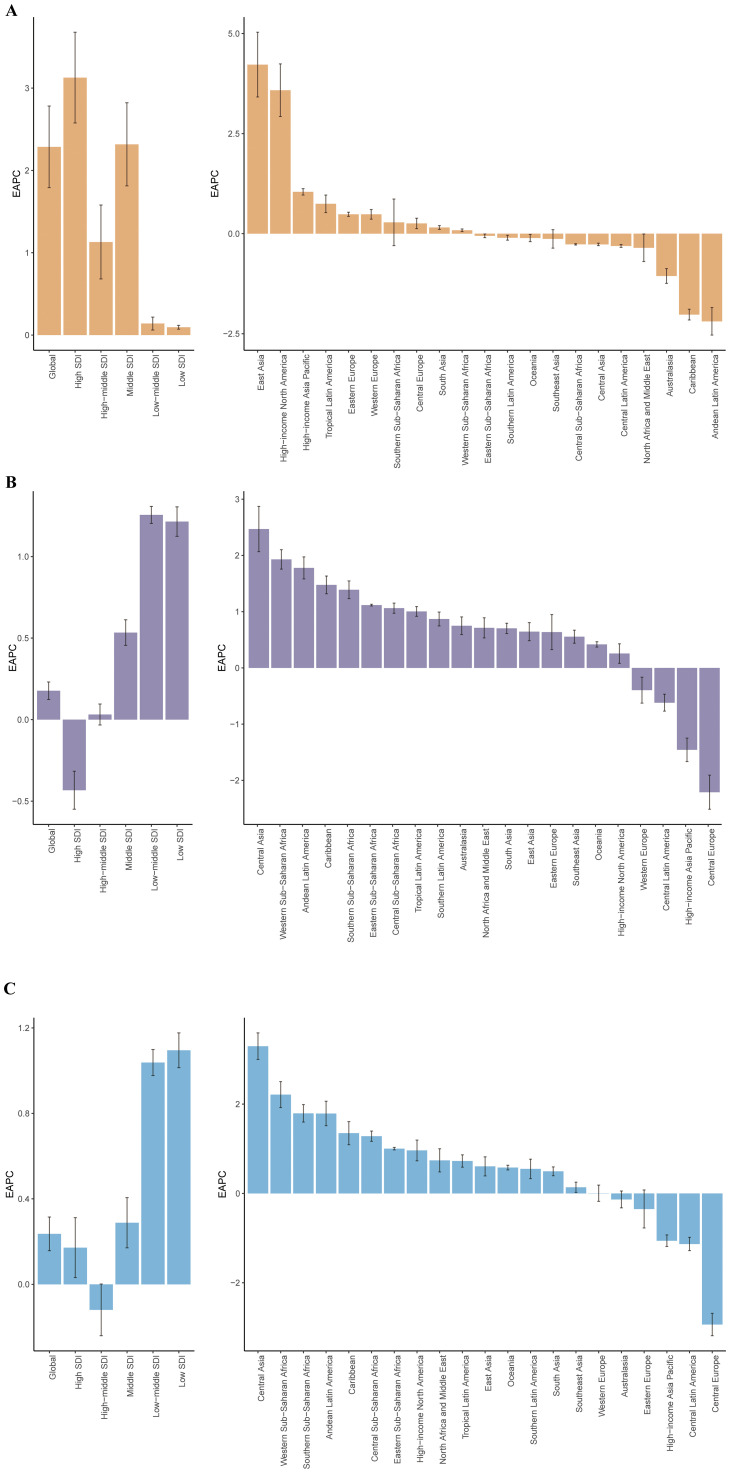
The EAPCs for ASPR **(A)**, ASMR **(B)**, and ASDR **(C)** due to cSCC from 1990 to 2021, for both sexes, across GBD regions and SDI quintiles. DALYs, disability-adjusted life-years; EAPC, estimated annual percentage change; GBD, Global Burden of Disease; SDI, Socio-demographic Index. ASPR, age-standardized prevalence rate; ASMR, age-standardized mortality rate; ASDR, age-standardized disability-adjusted life year rate; cSCC, cutaneous squamous cell carcinoma.

From 1990 to 2021, the ASPR burden of cSCC increased across all SDI regions, with the most substantial rise observed in the middle SDI region, marked by an EAPC of 3.13 (95% UI: 2.58–3.68). The ASMR also increased in most regions, with the largest growth in the low-middle SDI region, reaching an EAPC of 1.26 (95% UI: 1.20–1.31), while the high SDI region was the only one to experience a decrease, with an EAPC of -0.43 (95% UI: -0.55 to -0.32). In contrast, the ASDR generally rose across nearly all SDI regions except the high-middle SDI region, which saw a slight decline, with an EAPC of -0.12 (95% UI: -0.24 to 0.01) (**Figures 2A–C**, **3A–C**).

Throughout 2021, the ASPR for cSCC was continuously greatest in the high SDI region (89.98 per 100,000 people), followed by the middle SDI region (8.71 per 100,000 people), and the ASDR was greatest in the high SDI region (16.43 per 100,000 population), while the low SDI region had the lowest ASPR, ASMR and ASDR (**Table 1**).

### 3.3 Geographic Regional Burden of cSCC

In 2021, High-income North America reported the highest number of cSCC cases, with a prevalence of 1,830,711.15 cases (95% UI: 1,568,781.98 to 2,192,144.72) and an ASPR of 494.55 per 100,000 people. Australasia recorded the highest ASMR at 3.19 and the highest ASDR at 55.30 per 100,000. In contrast, Oceania registered the lowest ASPR at 0.04, while Western Sub-Saharan Africa had both the lowest ASDR at 3.71 and ASMR at 0.15 per 100,000 people.

**Figure 4:**
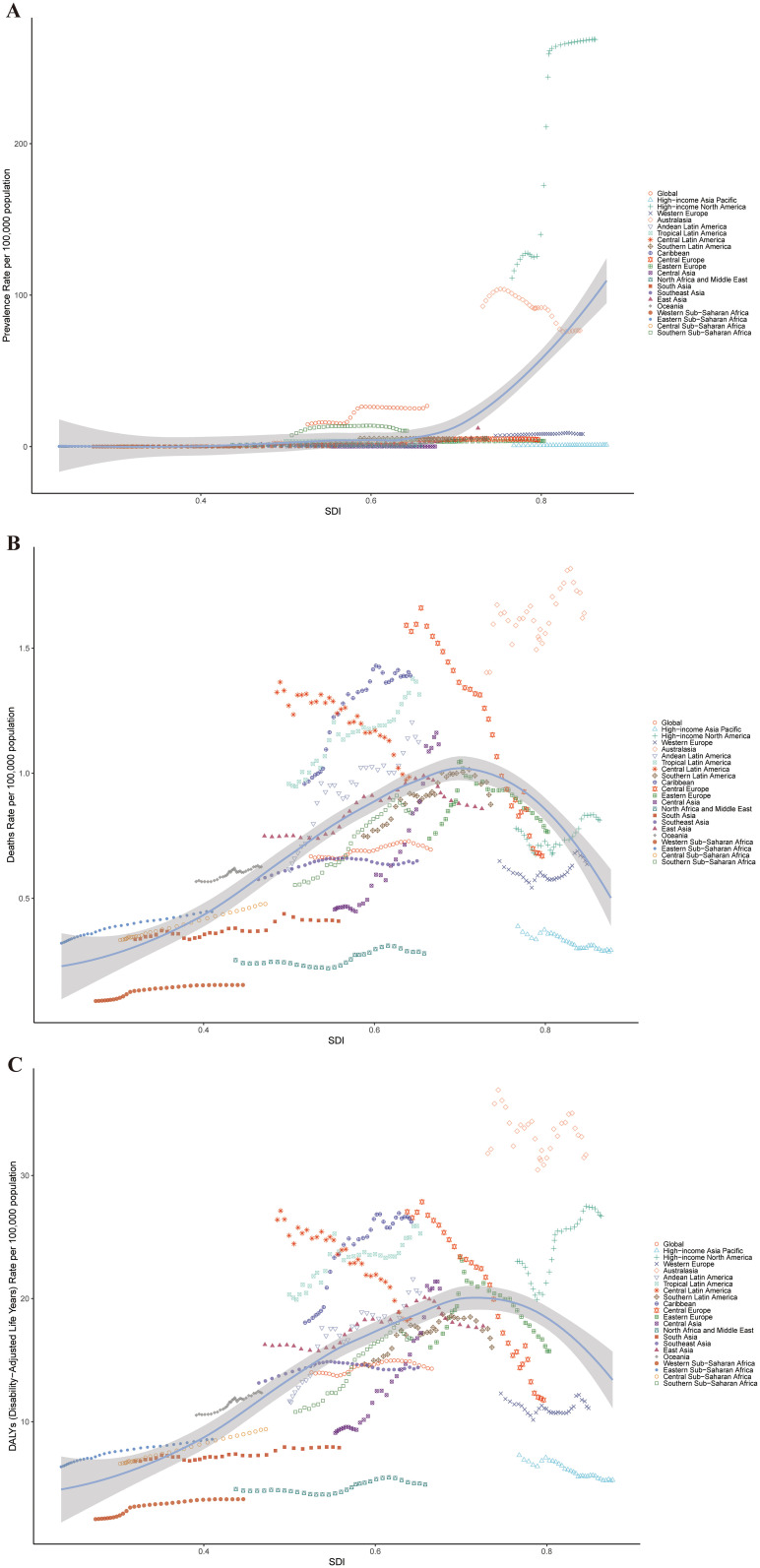
ASPR **(A)**, ASMR **(B)**, and ASDR **(C)** for cSCC across different geographical regions in 2021. ASPR, age-standardized prevalence rate; ASMR, age-standardized mortality rate; ASDR, age-standardized disability-adjusted life year rate; cSCC, cutaneous squamous cell carcinoma.

Across GBD regions from 1990 to 2021, the ASPR of cSCC increased in ten regions, with East Asia experiencing the largest rise (EAPC = 4.22) and Andean Latin America seeing the greatest decrease (EAPC = -2.19). For ASMR, it rose in seventeen GBD regions but declined in four, with Central Asia showing the highest increase (EAPC = 2.47) and Central Europe experiencing the largest reduction (EAPC = -2.21). Similarly, ASDR rose in sixteen regions and decreased in nine, with Central Asia having the most pronounced rise (EAPC = 3.29) and Central Europe recording the steepest decline (EAPC = -2.94) (**Figures 3A–C**, **4A–C**).

### 3.4 National burden of cSCC

In 2021, the United States recorded the highest number of cSCC cases, reaching 1,820,873, followed by China with 260,360 cases. The United States also reported the highest ASPR at 300.40 per 100,000 individuals. In Georgia, the highest ASMR and ASDR were observed, at 3.39 and 64.63 per 100,000 individuals, respectively. Conversely, Bermuda documented the lowest ASPR at 0.016 per 100,000 individuals, while Syria recorded the lowest ASMR and ASDR at 0.002 and 0.067 per 100,000 individuals, respectively (**Figures 1A–C**).

Between 1990 and 2021, the ASPR for cSCC increased in most countries. China experienced the most significant rise in ASPR, with an EAPC of 4.25. A similar trend was observed in the ASMR, with Georgia showing the largest increase at an EAPC of 9.78. Additionally, the ASDR rose in most countries, with Mongolia reporting the highest increase, also at an EAPC of 9.38.

### 3.5 Age-specific and sex burden of cSCC

**Figure 5:**
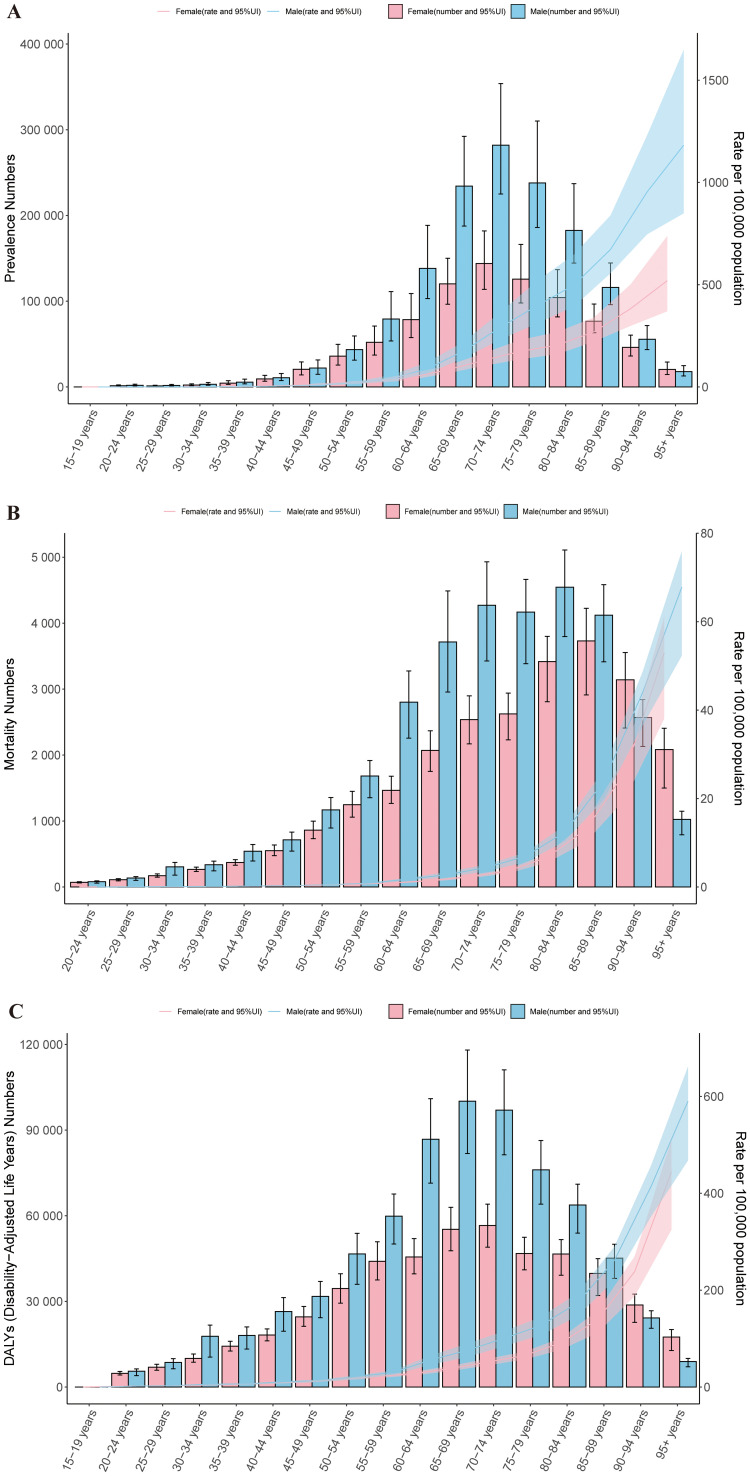
Age-specific patterns by sex for ASPR **(A)**, ASMR **(B)**, and ASDR **(C)** associated with cSCC at the global level in 2021. Error bars indicate the 95% uncertainty interval (UI) for the number of cases. Shading indicates the 95% UI for the rates. DALYs, disability-adjusted life-years. ASPR, age-standardized prevalence rate; ASMR, age-standardized mortality rate; ASDR, age-standardized disability-adjusted life year rate; cSCC, cutaneous squamous cell carcinoma.

In 2021, the prevalence of cSCC was primarily concentrated in the 55 to 94 age group. Among all age groups, the ASPR was higher in males than in females, except for the 90-94 age group. A similar pattern was observed in the ASMR. As age increased, the ASPR, ASMR, and ASDR all exhibited an upward trend. Notably, the ASPR, ASMR, and ASDR all reached their highest levels in the 95+ age group, with males consistently showing higher rates than females across these metrics (**Figures 5A–C**).

### 3.6 BAPC analysis

**Figure 6:**
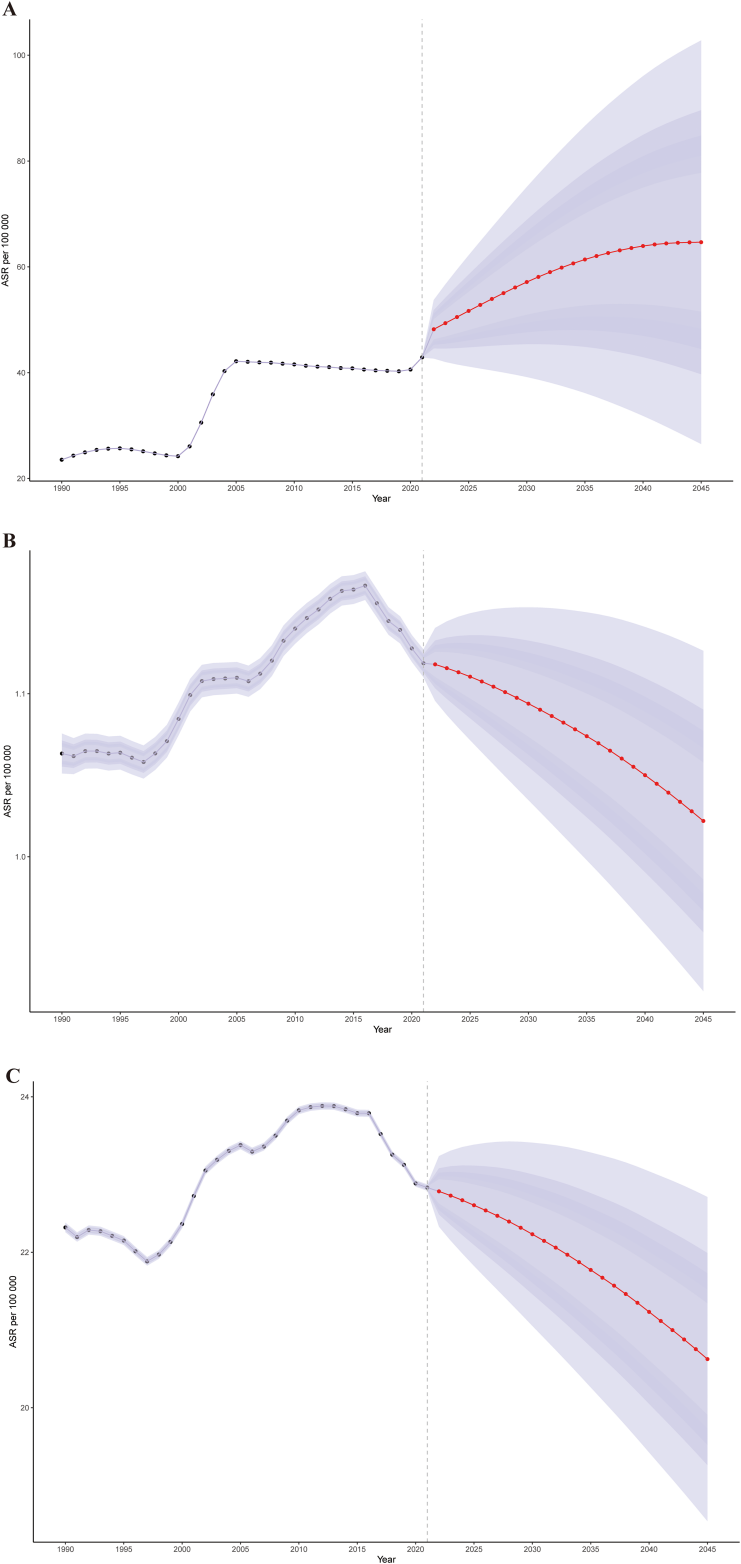
The global trends in ASPR **(A)**, ASMR **(B)**, and ASDR **(C)** from 2021 to 2045 for cSCC were predicted using Bayesian age-period-cohort (BAPC) models. ASPR, age-standardized prevalence rate; ASMR, age-standardized mortality rate; ASDR, age-standardized disability-adjusted life year rate; cSCC, cutaneous squamous cell carcinoma.

To analyze the trends in the ASPR, ASMR, and ASDR of cSCC after 2021, we used Bayesian age-period-cohort (BAPC) models to forecast these rates globally from 2021 to 2045. The projections suggest that the ASPR will increase from 42.89 per 100,000 in 2021 to 64.66 by 2045 (**Figure 6A**). In contrast, the ASMR is expected to decrease slightly from 1.12 to 1.02, while the ASDR is projected to drop from 22.83 to 20.63 over the same timeframe (**Figures 6B, C**). Moreover, the ASPR is anticipated to rise across most age groups, with the highest rate reaching 1661.59 per 100,000 among individuals over 95 years old by 2045.

### 3.7 Decomposition analysis

**Figure 7:**
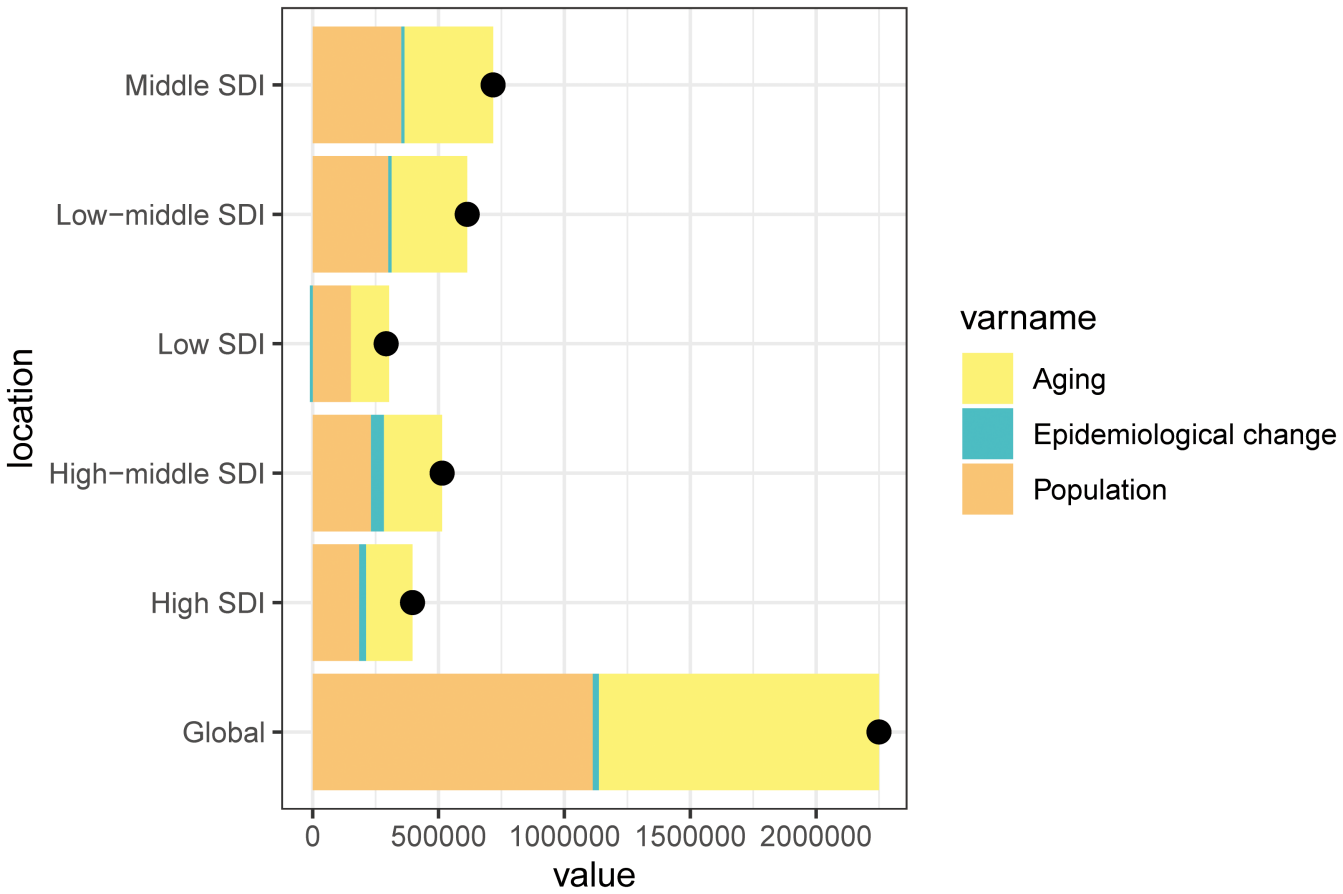
Changes in the ASMR of cSCC according to the three causes from 1990 to 2021 at the global level and by SDI quintile and WHO regions. The black dot represents the overall value of incidence change contributed from all causes. ASMR, age-standardized mortality rate; WHO, World Health Organization; cSCC, cutaneous squamous cell carcinoma.

We conducted a decomposition analysis to assess the trends in ASMR for cSCC globally and within five SDI regions. This analysis focused on the roles of population growth, demographic aging, and epidemiological changes. The global ASMR rate significantly increased, with the middle SDI region experiencing the highest rise, amounting to 716,627.41. From 1990 to 2021, the increase in mortality was driven by both population growth and aging, a pattern that was consistent across all SDI regions (**Figure 7**).

Among the SDI regions, population growth had the largest impact on changes in cSCC mortality from 1990 to 2021, particularly in the low SDI region (51.96%), followed by the middle SDI region (49.48%) and low-middle SDI region (48.94%). The contributions from the high SDI region (46.60%) and the high-middle SDI region (45.01%) were slightly lower. Similarly, population aging contributed positively to cSCC mortality, with the same order of contributions across the regions. In contrast, epidemiological changes had a positive impact in the high-middle SDI region (9.98%) and high SDI region (6.80%) while being negative in the low SDI region (−3.91%) (**Figure 7**).

### 3.8 Frontier analysis in DALY rate of cSCC

**Figure 8:**
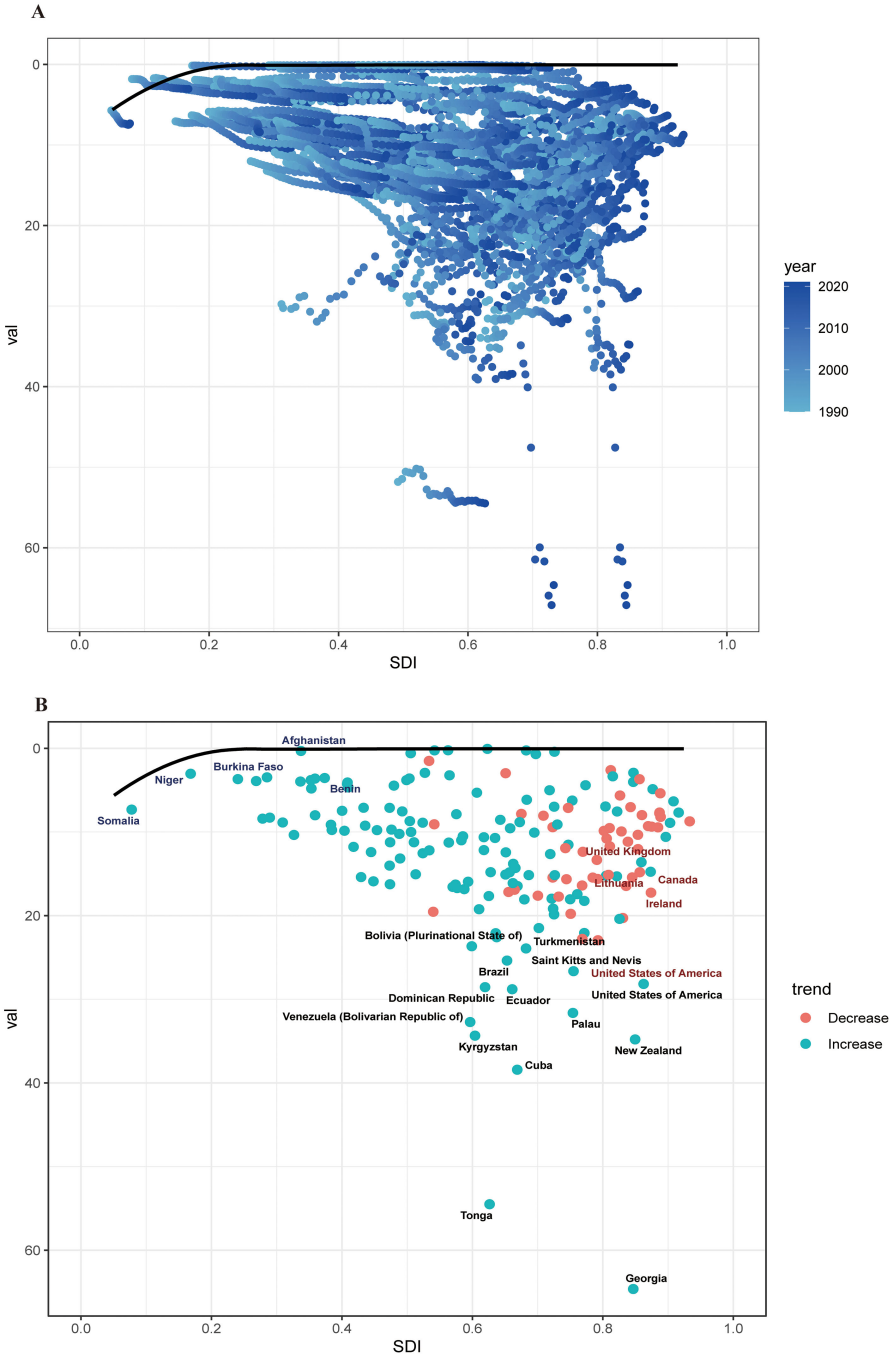
**(A)** Frontier analysis of cSCC based on SDI and ASDR from 1990 to 2021. The color scale represents the years from 1990, depicted in black, to 2021, shown in blue. The frontier is delineated in a solid black color. **(B)** Frontier analysis based on SDI and cSCC ASDR in 2021. The frontier line is black; countries and territories are represented as dots. The top countries with the most considerable effective difference of ASDR from the frontier line are marked in black words; Red dots indicate an increase in ASDR of cSCC from 1990 to 2021; blue dots indicate a decrease in ASDR of cSCC between 1990 and 2021. ASDR, age-standardized disability-adjusted life year rate; cSCC, cutaneous squamous cell carcinoma.

Using ASDR as a primary parameter, we performed a frontier analysis across 204 nations and regions from 1990 to 2021 in order to better understand changes in DALY rates linked to cSCC. Based on their SDI levels, countries were grouped along a borderline; the frontier denoted those with the lowest DALY rates in relation to their SDI. Optimizing sociodemographic resources can help close the “effective difference” between a country’s actual and attainable DALYs. Interestingly, the biggest effective disparities were seen in Georgia, Tonga, and Cuba, showing far greater ASDR when compared to comparably developed countries. On the other hand, in relation to their levels of development, the Syrian Arab Republic, Morocco, and Sudan had the lowest ASDR (**Figures 8A, B**).

### 3.9 Cross-country inequality analysis

**Figure 9:**
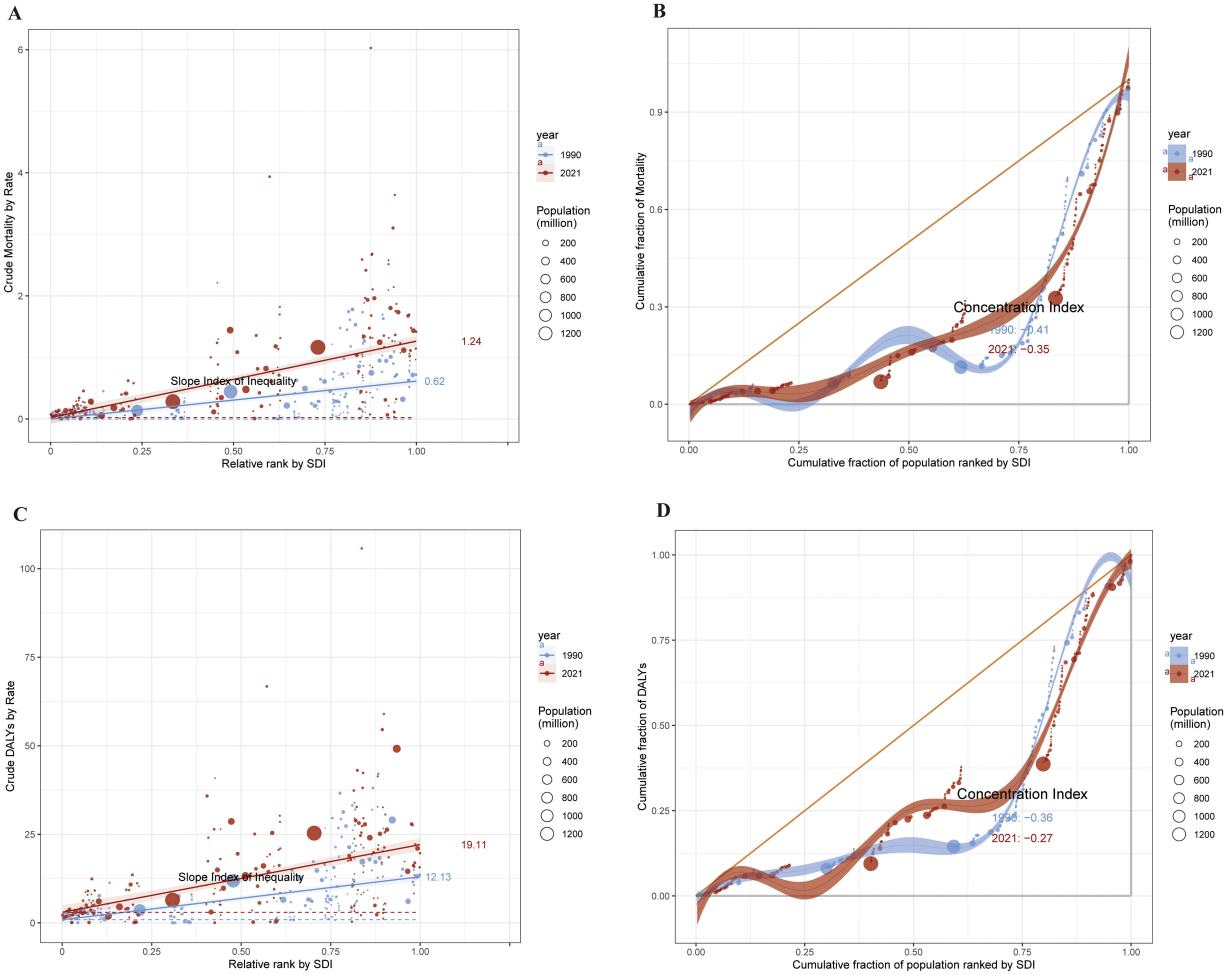
1990 and 2021, health inequality regression curves **(A)** and concentration curves **(B)** for ASMR of cSCC. Health inequality regression curves **(C)** and concentration curves **(D)** for ASDR of cSCC. ASMR, age-standardized mortality rate; ASDR, age-standardized disability-adjusted life year rate; cSCC, cutaneous squamous cell carcinoma.

We performed an SDI-related health inequality study to investigate the connection between socioeconomic status and the burden of cSCC. Inequalities related to SDI were found to be both absolute and relative. The burden of ASMR and ASDR was disproportionately higher in nations with more SDI. For ASMR, the relative concentration index shifted from -0.41 in 1990 to -0.35 in 2021, while the slope index showed variations between nations from 0.62(95% CI:0.49-0.75) in 1990 to 1.24(95% CI:1.05-1.44). For ASDR, the relative concentration index was -0.27 in 2021 and -0.36 in 1990, whereas the slope index varied from 12.12 (95% CI: 9.59 to 14.66) in 1990 to 19.11 (95% CI: 15.54 to 22.68) in 2021. Health disparities linked to ASMR and ASDR of cSCC associated with SDI persisted between 1990 and 2021(**Figures 9A–D**).

## 4 Discussion

Cutaneous squamous cell carcinoma (cSCC) has become a prominent global health issue, increasingly recognized in the context of public health research. Historically, most studies have focused on melanoma, leaving a significant gap in our understanding of non-melanoma skin cancers like cSCC (4). The development of SCC has been associated with a number of risk factors. The main risk factors for SCC include aging, exposure to UV light, and occupation hazards (32). Recent research indicates a rising incidence of cSCC, particularly in regions with UV exposure (33). The majority of mutations discovered in cSCC have a “UV signature,” and epidemiological and clinical data support cumulative lifetime exposure to ultraviolet radiation as the primary environmental carcinogen responsible for cSCC (34). In addition to UV, exposure to polycyclic aromatic hydrocarbons, arsenic, and ionizing radiation at work and in the environment are other major risk factors for cSCC (35). Additionally, due to innate flaws in the immune system or DNA repair pathways, some hereditary disorders, like xeroderma pigmentosum and epidermodysplasia verruciformis, enhance vulnerability to cSCC (36).This study builds on existing literature by employing data from the 2021 GBD survey, providing a comprehensive analysis of cSCC prevalence, mortality, and DALYs across 204 countries. Importantly, our study employs a variety of innovative analytical techniques, including decomposition analysis to disentangle the contributions of demographic changes, frontier analysis to assess healthcare performance relative to socio-economic factors, and Bayesian age-period-cohort modeling to clarify temporal trends and project future burdens of cSCC (30, 37, 38).

The worldwide burden of cSCC is still increasing in ASPR and ASDR, despite the fact that primary and secondary preventive strategies for the disease, such as UVR protection and early self-detection, have the potential to significantly lower morbidity and medical expenses of cSCC in particular areas and nations (39, 40). Our analysis reveals that, as of 2021, there were over 2.27 million cases of cSCC worldwide, reflecting a 345% increase since 1990. The ASPR rose from 14.69 to 26.85 per 100,000 individuals, while the ASMR experienced a modest increase from 0.67 to 0.69 per 100,000. In terms of DALYs, there was a striking 122% increase, rising from approximately 545,000 to over 1.21 million. The primary cause of the rise in ASPR of cSCC globally is most likely the worsening effects of population aging (41). Additionally, cSCC-related ASPR increased abruptly and dramatically in East Asia in the past three decades, where cSCC preventive and monitoring initiatives should be implemented to save healthcare expenditures and morbidity (42). The SCC mortality and DALYs in Central Asia increased at a disproportionately high rate from 1990 to 2021 compared to other parts of the world. It is believed that a large number of patients with SCC and other cancers in Central Asia who present with late-stage disease have restricted resources, a lower socioeconomic status, and a lack of accessibility to early identification and prompt therapy (14, 43). High-income North America recorded the highest ASPR in 2021, yet it also has a relatively low ASMR. Our analysis highlights that regions with high UV exposure, such as High-Income North America and Australasia, exhibit the highest prevalence rates of cSCC. This aligns with previous studies showing that UV radiation is the primary environmental carcinogen for cSCC (33, 34). This discrepancy can be attributed to several factors. First, individuals in North America generally have lighter skin, which increases their susceptibility to UV light. Darker-skinned populations demonstrate lower prevalence rates due to higher melanin levels, which provide enhanced protection against UV-induced damage. Additionally, a cultural tendency to seek sunbathing contributes to this increased exposure, resulting in a higher detection rate of skin tumors. Furthermore, this trend reflects the significant efforts made in high-income regions to promote early screening and treatment of these tumors (44, 45). Public health strategies aimed at reducing UV exposure, such as promoting sun protection measures and regulating indoor tanning, could significantly mitigate cSCC prevalence in these regions. While resource-poor regions must make up for their lack of medical resources through early screening(such as dermatoscopy), resource-rich regions (like North America) have reduced mortality rates through effective care management and individualized approaches, such as immunotherapy and photodynamic therapy (46–48). Australia ranked first in the world for both ASDR and ASMR of cSCC in 2021. However, due to its national emphasis on lifelong outdoor activity, coupled with inadequate sun protection measures and a population with fair skin, Australia could benefit from North America’s approach. Specifically, Australia should focus on educating the public about effective sun protection, reducing outdoor exercise time during peak UV hours, and improving early diagnosis and treatment of skin lesions (49–51). By reducing the prevalence of indoor tanning in North America and Europe, prohibiting indoor tanning for the next generation of people between the ages of 12 and 35 might prevent 9.7 million keratinocyte carcinomas and 448,000 melanomas, as well as save US $5.7 billion in medical expenses (44).

Our findings reveal notable differences in cSCC prevalence and outcomes across ethnic groups. Darker-skinned populations (such as Africans and People of African Descent) demonstrate lower prevalence rates due to higher melanin levels, which provide enhanced protection against UV-induced damage. Conversely, lighter-skinned individuals (like Caucasoid), particularly in high-SDI regions, face higher risks due to their lower natural UV protection (52). This disparity underscores the need for targeted prevention strategies that account for genetic and environmental factors. For example, early screening and public education campaigns could help bridge the gap in outcomes between different ethnic groups and regions.

Our data also indicated that males had higher ASPR, ASMR, and ASDR compared to females, with the most pronounced disparities occurring in older age groups. This aligns with existing literature that highlights the male gender as a risk factor for cSCC due to greater UV exposure from outdoor activities, less sun protection, and occupational hazards (53, 54). The aging population intensifies these trends as older individuals experience heightened vulnerability to skin cancers. This is largely due to cumulative UV exposure over the years and a decline in immune function associated with aging, which compromises the body’s ability to detect and respond to malignancies (55, 56).

Between 1990 and 2021, the ASMR and ASPR of cSCC gradually increased globally. High SDI areas and affluent nations like America, Australasia, and New Zealand, where a significant section of the population is light-skinned, continue to have high prevalence and significant ASDR burdens from skin malignancies (57, 58). A major contributing factor to cSCC, aside from UVR exposure, is the correlation between skin color and skin cancer (59, 60). Dark-pigmented individuals have a lower incidence of skin cancer than light-colored Caucasians because their complexion has more epidermal melanin to defend it from UVR ray damage (52). Furthermore, using the global heat map, we were able to see regional differences in the incidence of cSCC, with larger incidence trends reported distant from the equator. However, it is important to note that the growth rate of ASMR is smaller than that of ASPR. This trend reflects the global efforts in recent years to enhance early screening, diagnosis, and treatment of skin cancer. Australia’s “Slip, Slop, Slap, Seek, and Slide” campaign has significantly reduced UV-related skin cancer by promoting sunscreen use, protective clothing, and limited sun exposure during peak hours (49). Similar programs in high-risk regions could mitigate rising cSCC cases. Outdoor workers, such as farmers and construction workers, are highly exposed to UV radiation. Preventative measures like workplace regulations, UV-protective gear, and sunscreen distribution have proven effective and should be implemented more broadly. Community-based screenings, like those by the American Academy of Dermatology, improve early cSCC detection, reducing morbidity and mortality (39). Expanding these programs to low- and middle-SDI regions is essential. Advances in teledermatology and AI (artificial intelligence)-powered diagnostic tools offer promising solutions for improving access to early detection in underserved populations. Advancements in machine learning (ML) have revolutionized skin cancer detection (61). Deep learning models, such as convolutional neural networks (CNNs), achieve high accuracy in classifying dermoscopic images, sometimes surpassing dermatologists (62, 63). Combined with imaging modalities like high-resolution dermoscopy, reflectance confocal microscopy (RCM), and optical coherence tomography (OCT), ML enables non-invasive, accurate cSCC detection, reducing unnecessary biopsies and supporting early intervention (61, 64). Expanding these tools to resource-limited regions could significantly reduce the global cSCC burden. These technologies facilitate remote evaluation of skin lesions and reduce the burden on specialized healthcare facilities. Pilot programs in regions with limited dermatology services have demonstrated their effectiveness (65, 66). Moreover, initiatives such as themed skincare days offering free diagnosis, increased patient awareness about outdoor exercise and sun protection, and public education on skin tumors can also have contributed to these improvements (67–70).

Compared to prior epidemiological studies, our analysis offers new insights by employing advanced methodologies. Earlier studies primarily reported rising cSCC prevalence but lacked global data or advanced modeling. For example, Zhang et al (2). demonstrated increasing cSCC incidence globally but relied heavily on descriptive statistics without exploring the contributions of demographic or epidemiological factors. Our analysis of cSCC trends using BAPC models, we projected the ASPR of cSCC to increase from 42.89 per 100,000 in 2021 to 64.66 per 100,000 by 2045, primarily due to demographic shifts, especially in aging populations. Unlike prior studies, the BAPC model allowed us to disentangle the specific impacts of age, period, and cohort on prevalence trends. Our analysis also revealed significant regional disparities, with high-SDI regions such as North America and Australasia experiencing the highest prevalence, likely due to UV exposure and higher rates of early detection. Conversely, ASMR and ASDR are projected to decline slightly, reflecting possible improvements in healthcare access and interventions aimed at early diagnosis and treatment (71). The anticipated rise in ASPR across most age groups, particularly among those over 95, underscores the need for targeted healthcare strategies to address the demands of an aging population (72).

Mortality trends in cSCC have been underreported in the literature, with most studies attributing rising death rates to late diagnoses or healthcare inequalities. For example, Gordon et al (52). noted a correlation between advanced age and higher cSCC mortality but lacked data on how demographic changes contributed to this increase. The decomposition analysis reveals that population growth and aging were primary contributors to increased ASMR across all SDI regions from 1990 to 2021, especially in low to middle-SDI region, where resource constraints may exacerbate the burden. This nuanced analysis showed that population aging was the dominant factor driving increased mortality, especially in low- and middle-SDI regions. Effective strategies, such as increasing awareness and screening in high-risk demographics, should be prioritized in these regions to mitigate further mortality increases (38).

Few studies have focused on the DALYs associated with cSCC. Studies such as Huang et al. (2024) emphasized DALYs for melanoma but offered limited data on cSCC (4). Frontier analysis in ASDR highlights significant effective disparities among countries, with regions like Georgia, Tonga, and Cuba experiencing higher-than-expected ASDR relative to their development levels. Conversely, countries like the Syrian Arab Republic and Sudan demonstrated lower ASDR rates, indicating potential resilience factors or effective healthcare strategies that could be emulated (73, 74). The use of frontier analysis allowed us to identify regions with disproportionate d healthcare system performance and resource allocation. Addressing these disparities by optimizing resource allocation and increasing preventive measures could narrow the “effective difference” in DALY rates globally.

Our findings from the cross-country inequality analysis underscore persistent health disparities in cSCC burden associated with SDI levels, which remained substantial from 1990 to 2021. The concentration and slope indexes indicate that higher SDI nations continue to face disproportionately higher ASMR and ASDR, likely due to differences in healthcare infrastructure and access. Policymakers should prioritize equitable access to healthcare resources, especially for under-resourced regions, to reduce these disparities and improve outcomes.

Several limitations of our study should be acknowledged. First, the GBD 2021 assessment lacks detailed information on skin cancer histology subtypes, risk factors, and specific mortality data for cSCC. This absence restricts our ability to analyze spatial and temporal trends in prevalence, DALYs, and mortality rates, which are crucial for understanding the disease. Second, the quality of the GBD data on cSCC raises concerns, particularly regarding underreporting. Variations in data sources and differing definitions of skin cancers can lead to inconsistencies that compromise the reliability of our findings. Lastly, our predictions did not account for external influences, such as changes in healthcare policy or public health initiatives, which could significantly impact the burden of skin cancer. Future research should address these limitations by incorporating more comprehensive data and considering these external factors to achieve a clearer understanding of skin cancer epidemiology.

## 5 Conclusions

In conclusion, the significant rise in cSCC prevalence and mortality reflects broader public health challenges linked to demographic shifts, environmental factors, and disparities in healthcare access. Addressing these issues requires a multifaceted approach focused on prevention, early detection, and equitable healthcare access. The findings underscore the urgent need for comprehensive strategies to manage and mitigate the burden of cSCC globally.

## Data Availability

The original contributions presented in the study are included in the article/supplementary material. Further inquiries can be directed to the corresponding author/s.

## References

[1] Guy GP Jr Machlin SR Ekwueme DU Yabroff KR. Prevalence and costs of skin cancer treatment in the U.S., 2002-2006 and 2007-2011. Am J Prev Med. (2015) 48:183–7. doi: 10.1016/j.amepre.2014.08.036 PMC4603424 25442229

[2] Zhang W Zeng W Jiang A He Z Shen X Dong X. Global, regional and national incidence, mortality and disability-adjusted life-years of skin cancers and trend analysis from 1990 to 2019: An analysis of the Global Burden of Disease Study 2019. Cancer Med. (2021) 10:4905–22. doi: 10.1002/cam4.4046 PMC8290243 34105887

[3] Karia PS Han J Schmults CD. Cutaneous squamous cell carcinoma: estimated incidence of disease, nodal metastasis, and deaths from disease in the United States, 2012. J Am Acad Dermatol. (2013) 68:957–66. doi: 10.1016/j.jaad.2012.11.037 23375456

[4] Huang S Jiang J Wong HS Zhu P Ji X Wang D. Global burden and prediction study of cutaneous squamous cell carcinoma from 1990 to 2030: A systematic analysis and comparison with China. J Glob Health. (2024) 14:4093. doi: 10.7189/jogh.14.04093 PMC11063968 38695259

[5] Elliott BM Douglass BR McConnell D Johnson B Harmston C. Incidence, demographics and surgical outcomes of cutaneous squamous cell carcinoma diagnosed in Northland, New Zealand. N Z Med J. (2018) 131:61–8. 29771903

[6] Lomas A Leonardi-Bee J Bath-Hextall F. A systematic review of worldwide incidence of nonmelanoma skin cancer. Br J Dermatol. (2012) 166:1069–80. doi: 10.1111/j.1365-2133.2012.10830.x 22251204

[7] Sung H Ferlay J Siegel RL Laversanne M Soerjomataram I Jemal A. Global cancer statistics 2020: GLOBOCAN estimates of incidence and mortality worldwide for 36 cancers in 185 countries. CA Cancer J Clin. (2021) 71:209–49. doi: 10.3322/caac.21660 33538338

[8] Aggarwal P Knabel P Fleischer AB Jr. United States burden of melanoma and non-melanoma skin cancer from 1990 to 2019. J Am Acad Dermatol. (2021) 85:388–95. doi: 10.1016/j.jaad.2021.03.109 33852922

[9] Seidl S Schuster B Rüth M Biedermann T Zink A. What do germans want to know about skin cancer? A nationwide google search analysis from 2013 to 2017. J Med Internet Res. (2018) 20:e10327. doi: 10.2196/10327 29698213 PMC5956155

[10] Jiang R Fritz M Que S. Cutaneous squamous cell carcinoma: an updated review. Cancers (Basel). (2024) 16:1800. doi: 10.3390/cancers16101800 38791879 PMC11119634

[11] Huang J Chan SC Ko S Lok V Zhang L Lin X. Global incidence, mortality, risk factors and trends of melanoma: A systematic analysis of registries. Am J Clin Dermatol. (2023) 24:965–75. doi: 10.1007/s40257-023-00795-3 37296344

[12] Cao X Wang M Zhou M Mi Y Guo Q Fan Y. Global, regional and national burden of paediatric atopic dermatitis: A trend and geographic inequalities analysis. Clin Exp Allergy. (2024) 54:747–59. doi: 10.1111/cea.14558 39179382

[13] GBD 2019 IMID Collaborators. Global, regional, and national incidence of six major immune-mediated inflammatory diseases: findings from the global burden of disease study 2019. EClinicalMedicine. (2023) 64:102193. doi: 10.1016/j.eclinm.2023.102193 37731935 PMC10507198

[14] Urban K Chu S Giesey RL Mehrmal S Uppal P Delost ME. Burden of skin disease and associated socioeconomic status in Asia: A cross-sectional analysis from the Global Burden of Disease Study 1990-2017. JAAD Int. (2021) 2:40–50. doi: 10.1016/j.jdin.2020.10.006 34409353 PMC8362322

[15] GBD 2021 Diseases and Injuries Collaborators. Global incidence, prevalence, years lived with disability (YLDs), disability-adjusted life-years (DALYs), and healthy life expectancy (HALE) for 371 diseases and injuries in 204 countries and territories and 811 subnational locations, 1990-2021: a systematic analysis for the Global Burden of Disease Study 2021. Lancet. (2024) 403:2133–61. doi: 10.1016/S0140-6736(24)00757-8 PMC11122111 38642570

[16] Lv B Lan JX Si YF Ren YF Li MY Guo FF. Epidemiological trends of subarachnoid hemorrhage at global, regional, and national level: a trend analysis study from 1990 to 2021. Mil Med Res. (2024) 11:46. doi: 10.1186/s40779-024-00551-6 38992778 PMC11241879

[17] Jang H Park S Kim MS Yon DK Lee SW Koyanagi A. Global, regional and national burden of alopecia areata and its associated diseases, 1990-2019: A systematic analysis of the Global Burden of Disease Study 2019. Eur J Clin Invest. (2023) 53:e13958. doi: 10.1111/eci.13958 36692126

[18] Zhang Y Dong S Ma Y Mou Y. Burden of psoriasis in young adults worldwide from the global burden of disease study 2019. Front Endocrinol (Lausanne). (2024) 15:1308822. doi: 10.3389/fendo.2024.1308822 38414821 PMC10897041

[19] GBD 2019 Demographics Collaborators. Global age-sex-specific fertility, mortality, healthy life expectancy (HALE), and population estimates in 204 countries and territories, 1950-2019: a comprehensive demographic analysis for the Global Burden of Disease Study 2019. Lancet. (2020) 396:1160–203. doi: 10.1016/S0140-6736(20)30977-6 PMC7566045 33069325

[20] Jackson JW VanderWeele TJ. Intersectional decomposition analysis with differential exposure, effects, and construct. Soc Sci Med. (2019) 226:254–9. doi: 10.1016/j.socscimed.2019.01.033 PMC6886261 30770131

[21] Cheng X Yang Y Schwebel DC Liu Z Li L Cheng P. Population ageing and mortality during 1990-2017: A global decomposition analysis. PLoS Med. (2020) 17:e1003138. doi: 10.1371/journal.pmed.1003138 32511229 PMC7279585

[22] Jamieson L Mejia G Luzzi L Ju X. Oral health inequities among CALD and non-CALD older Australians: A decomposition analysis. Int J Environ Res Public Health. (2023) 20:6455. doi: 10.3390/ijerph20156455 37568999 PMC10418650

[23] Ruan R Liu X Zhang Y Tang M He B Zhang QW. Global, regional, and national advances toward the management of rheumatic heart disease based on the global burden of disease study 2019. J Am Heart Assoc. (2023) 12:e028921. doi: 10.1161/JAHA.122.028921 37366108 PMC10356074

[24] Bai Z Han J An J Wang H Du X Yang Z. The global, regional, and national patterns of change in the burden of congenital birth defects, 1990-2021: an analysis of the global burden of disease study 2021 and forecast to 2040. EClinicalMedicine. (2024) 77:102873. doi: 10.1016/j.eclinm.2024.102873 39416384 PMC11474384

[25] Zhang Y Feng L Zhu Z He Y Li X. Global burden of myocarditis in youth and middle age (1990-2019): A systematic analysis of the disease burden and thirty-year forecast. Curr Probl Cardiol. (2024) 49:102735. doi: 10.1016/j.cpcardiol.2024.102735 38950720

[26] Lin Y Jiang B Cai Y Luo W Zhu X Lin Q. The global burden of glaucoma: findings from the global burden of disease 2019 study and predictions by bayesian age-period-cohort analysis. J Clin Med. (2023) 12:1828. doi: 10.3390/jcm12051828 36902615 PMC10003840

[27] Maimaiti A Tuersun M Wang X Mijiti M Wu H Song C. Global, regional, and national burden of brain and central nervous system cancers for males from 1990 to 2021 and its predicted level in the next 25 years. Neuroepidemiology. (2024), 1–22. doi: 10.1159/000541917 39447550

[28] Li J Jia H Liu Z Xu K. Global, regional and national trends in the burden of low bone mineral density from 1990 to 2030: A Bayesian age-period-cohort modeling study. Bone. (2024) 189:117253. doi: 10.1016/j.bone.2024.117253 39245331

[29] Hu C Ding L Peng K. Global burden of major depressive disorders attributable to intimate partner violence against women: Magnitude, temporal trends, and regional inequalities. J Affect Disord. (2024) 363:182–91. doi: 10.1016/j.jad.2024.07.098 39025448

[30] Liu Y Yao S Shan X Luo Y Yang L Dai W. Time trends and advances in the management of global, regional, and national diabetes in adolescents and young adults aged 10-24 years, 1990-2021: analysis for the global burden of disease study 2021. Diabetol Metab Syndr. (2024) 16:252. doi: 10.1186/s13098-024-01491-w 39456070 PMC11515246

[31] Ordunez P Martinez R Soliz P Giraldo G Mujica OJ Nordet P. Rheumatic heart disease burden, trends, and inequalities in the Americas, 1990-2017: a population-based study. Lancet Glob Health. (2019) 7:e1388–97. doi: 10.1016/S2214-109X(19)30360-2 31537369

[32] Comune R Ruggiero A Portarapillo A Villani A Megna M Tamburrini S. Cutaneous squamous cell carcinoma: from diagnosis to follow-up. Cancers (Basel). (2024) 16:2960. doi: 10.3390/cancers16172960 39272818 PMC11394133

[33] Armstrong BK Kricker A. The epidemiology of UV induced skin cancer. J Photochem Photobiol B. (2001) 63:8–18. doi: 10.1016/s1011-1344(01)00198-1 11684447

[34] Inman GJ Wang J Nagano A Alexandrov LB Purdie KJ Taylor RG. The genomic landscape of cutaneous SCC reveals drivers and a novel azathioprine associated mutational signature. Nat Commun. (2018) 9:3667. doi: 10.1038/s41467-018-06027-1 30202019 PMC6131170

[35] Kong YH Xu SP. Salidroside prevents skin carcinogenesis induced by DMBA/TPA in a mouse model through suppression of inflammation and promotion of apoptosis. Oncol Rep. (2018) 39:2513–26. doi: 10.3892/or.2018.6381 PMC5983924 29693192

[36] Leiter U Keim U Garbe C. Epidemiology of skin cancer: update 2019. Adv Exp Med Biol. (2020) 1268:123–39. doi: 10.1007/978-3-030-46227-7_6 32918216

[37] Pan H Zhao Z Deng Y Zheng Z Huang Y Huang S. The global, regional, and national early-onset colorectal cancer burden and trends from 1990 to 2019: results from the Global Burden of Disease Study 2019. BMC Public Health. (2022) 22:1896. doi: 10.1186/s12889-022-14274-7 36221047 PMC9555189

[38] Huang K Huang X Qian S Cai Y Wu F Luo D. Temporal trends of thyroid cancer in China and globally from 1990 to 2021: an analysis of the global burden of Disease Study 2021. Sci Rep. (2024) 14:25538. doi: 10.1038/s41598-024-77663-5 39462100 PMC11513994

[39] Okhovat JP Beaulieu D Tsao H Halpern AC Michaud DS Shaykevich S. The first 30 years of the American Academy of Dermatology skin cancer screening program: 1985-2014. J Am Acad Dermatol. (2018) 79:884–91.e3. doi: 10.1016/j.jaad.2018.05.1242 30057360 PMC6454210

[40] Gao YS Lai DH Cheng SW Li Q Hao JC. Investigation on the awareness and behavior of primary school students on sunscreen use in beijing. Clin Cosmet Investig Dermatol. (2022) 15:887–94. doi: 10.2147/CCID.S365856 PMC9121882 35601539

[41] Wysong A. Squamous-cell carcinoma of the skin. N Engl J Med. (2023) 388:2262–73. doi: 10.1056/NEJMra2206348 37314707

[42] Gordon LG Elliott TM Wright CY Deghaye N Visser W. Modelling the healthcare costs of skin cancer in South Africa. BMC Health Serv Res. (2016) 16:113. doi: 10.1186/s12913-016-1364-z 27039098 PMC4818961

[43] Bray F Laversanne M Sung H Ferlay J Siegel RL Soerjomataram I. Global cancer statistics 2022: GLOBOCAN estimates of incidence and mortality worldwide for 36 cancers in 185 countries. CA Cancer J Clin. (2024) 74:229–63. doi: 10.3322/caac.21834 38572751

[44] Gordon LG Rodriguez-Acevedo AJ Køster B Guy GP Jr Sinclair C Van Deventer E. Association of indoor tanning regulations with health and economic outcomes in North America and Europe. JAMA Dermatol. (2020) 156:401–10. doi: 10.1001/jamadermatol.2020.0001 PMC7042819 32074257

[45] Walkosz BJ Scott MD Buller DB Andersen PA Beck L Cutter GR. Prevalence of Sun Protection at Outdoor Recreation and Leisure Venues at Resorts in North America. Am J Health Educ. (2017) 48:90–9. doi: 10.1080/19325037.2016.1271755 PMC5683731 29147456

[46] Work Group Invited Reviewers Kim J Kozlow JH Mittal B Moyer J. Guidelines of care for the management of cutaneous squamous cell carcinoma. J Am Acad Dermatol. (2018) 78:560–78. doi: 10.1016/j.jaad.2017.10.007 PMC6652228 29331386

[47] Schmults CD Blitzblau R Aasi SZ Alam M Andersen JS Baumann BC. NCCN guidelines® Insights: squamous cell skin cancer, version 1. 2022. J Natl Compr Canc Netw. (2021) 19:1382–94. doi: 10.6004/jnccn.2021.0059 34902824

[48] Stratigos AJ Garbe C Dessinioti C Lebbe C van Akkooi A Bataille V. European consensus-based interdisciplinary guideline for invasive cutaneous squamous cell carcinoma: Part 2. Treatment-Update 2023. Eur J Cancer. (2023) 193:113252. doi: 10.1016/j.ejca.2023.113252 37708630

[49] Morton SK Harrison SL. Slip, slop, slap, slide, seek and sport: A systematic scoping review of sun protection in sport in Australasia. Curr Oncol. (2022) 30:401–15. doi: 10.3390/curroncol30010033 PMC9858120 36661682

[50] Gao DX Swetter SM Hawryluk EB Geller AC Beaulieu D. Screening motivations among participants of the American Academy of Dermatology's SPOT Skin Cancer screening program from 2018 to 2019: A cross-sectional analysis. J Am Acad Dermatol. (2023) 88:674–6. doi: 10.1016/j.jaad.2022.06.1194 35803403

[51] Kolitz E Lopes F Arffa M Pineider J Bogucka R Adamson AS. UV exposure and the risk of keratinocyte carcinoma in skin of color: A systematic review. JAMA Dermatol. (2022) 158:542–6. doi: 10.1001/jamadermatol.2022.0263 PMC8943625 35319719

[52] Gordon R. Skin cancer: an overview of epidemiology and risk factors. Semin Oncol Nurs. (2013) 29:160–9. doi: 10.1016/j.soncn.2013.06.002 23958214

[53] Climstein M Doyle B Stapelberg M Rosic N Hertess I Furness J. Point prevalence of non-melanoma and melanoma skin cancers in Australian surfers and swimmers in Southeast Queensland and Northern New South Wales. PeerJ. (2022) 10:e13243. doi: 10.7717/peerj.13243 35505675 PMC9057286

[54] Eden M Hainsworth R Gordon LG Epton T Lorigan P Rhodes LE. Cost-effectiveness of a policy-based intervention to reduce melanoma and other skin cancers associated with indoor tanning. Br J Dermatol. (2022) 187:105–14. doi: 10.1111/bjd.21046 PMC9541204 35141876

[55] Huang J Zhang L Shi L Wu M Lv T Zhang Y. An epidemiological study on skin tumors of the elderly in a community in Shanghai, China. Sci Rep. (2023) 13:4441. doi: 10.1038/s41598-023-29012-1 36932111 PMC10023674

[56] Tseng HW Shiue YL Tsai KW Huang WC Tang PL Lam HC. Risk of skin cancer in patients with diabetes mellitus: A nationwide retrospective cohort study in Taiwan. Med (Baltimore). (2016) 95:e4070. doi: 10.1097/MD.0000000000004070 PMC4937962 27368048

[57] Paul S Chen Y Mohaghegh M. Analysis of prevalence, socioeconomic and disease trends of non-melanoma skin cancer in New Zealand from 2008 to 2022. J Epidemiol Glob Health. (2024) 14:1012–21. doi: 10.1007/s44197-024-00250-4 PMC11442425 38842790

[58] Conte S Aldien AS Jetté S LeBeau J Alli S Netchiporouk E. Skin cancer prevention across the G7, Australia and New Zealand: A review of legislation and guidelines. Curr Oncol. (2023) 30:6019–40. doi: 10.3390/curroncol30070450 PMC10377770 37489567

[59] Armstrong BK Cust AE. Sun exposure and skin cancer, and the puzzle of cutaneous melanoma: A perspective on Fears et al. Mathematical models of age and ultraviolet effects on the incidence of skin cancer among whites in the United States. Am J Epidemiol. (1977) 105:420–7. doi: 10.1016/j.canep.2017.04.004 860705

[60] Rass K Reichrath J. UV damage and DNA repair in Malignant melanoma and nonmelanoma skin cancer. Adv Exp Med Biol. (2008) 624:162–78. doi: 10.1007/978-0-387-77574-6_13 18348455

[61] Esteva A Kuprel B Novoa RA Ko J Swetter SM Blau HM. Dermatologist-level classification of skin cancer with deep neural networks. Nature. (2017) 542:115–8. doi: 10.1038/nature21056 PMC8382232 28117445

[62] Chaahat AH Kumar Gondhi N Kumar Lehana P. An evolutionary approach for the enhancement of dermatological images and their classification using deep learning models. J Healthc Eng. (2021) 2021:8113403. doi: 10.1155/2021/8113403 34326979 PMC8302402

[63] Gareau DS Browning J Correa Da Rosa J Suarez-Farinas M Lish S Zong AM. Deep learning-level melanoma detection by interpretable machine learning and imaging biomarker cues. J BioMed Opt. (2020) 25:112906. doi: 10.1117/1.JBO.25.11.112906 33247560 PMC7702097

[64] Huang HY Hsiao YP Mukundan A Tsao YM Chang WY Wang HC. Classification of skin cancer using novel hyperspectral imaging engineering via YOLOv5. J Clin Med. (2023) 12:1134. doi: 10.3390/jcm12031134 36769781 PMC9918106

[65] Felmingham C MacNamara S Cranwell W Williams N Wada M Adler NR. Improving Skin cancer Management with ARTificial Intelligence (SMARTI): protocol for a preintervention/postintervention trial of an artificial intelligence system used as a diagnostic aid for skin cancer management in a specialist dermatology setting. BMJ Open. (2022) 12:e050203. doi: 10.1136/bmjopen-2021-050203 PMC8728443 34983756

[66] Uppal SK Beer J Hadeler E Gitlow H Nouri K. The clinical utility of teledermoscopy in the era of telemedicine. Dermatol Ther. (2021) 34:e14766. doi: 10.1111/dth.14766 33421232

[67] Yip WC Hsiao WC Chen W Hu S Ma J Maynard A. Early appraisal of China's huge and complex health-care reforms. Lancet. (2012) 379:833–42. doi: 10.1016/S0140-6736(11)61880-1 22386036

[68] Blumenthal D Hsiao W. Privatization and its discontents–the evolving Chinese health care system. N Engl J Med. (2005) 353:1165–70. doi: 10.1056/NEJMhpr051133 16162889

[69] Wan M Hu R Li Y Wang Y Xie X Yue P. Attitudes, beliefs, and measures taken by parents to protect their children from the sun in Guangzhou City, China. Photochem Photobiol. (2016) 92:753–9. doi: 10.1111/php.12623 27463620

[70] McNoe BM Gage R Signal L. What can Aotearoa New Zealand learn from the Australian Sunsmart Story? A qualitative study. Aust N Z J Public Health. (2022) 46:387–93. doi: 10.1111/1753-6405.13243 35436015

[71] Guo R Hou M Han Y Feng XL. Access, charge and quality of tele-dermatology e-consults in China: A standardized patients study. Digit Health. (2022) 8:20552076221140763. doi: 10.1177/20552076221140763 36465986 PMC9716584

[72] Fang EF Xie C Schenkel JA Wu C Long Q Cui H. A research agenda for ageing in China in the 21st century (2nd edition): Focusing on basic and translational research, long-term care, policy and social networks. Ageing Res Rev. (2020) 64:101174. doi: 10.1016/j.arr.2020.101174 32971255 PMC7505078

[73] Maloney ME Miranda-Galvis M Juarez BS Mamouni K Odhiambo L Ibrahim S. Teledermatology for skin cancer screening in rural Georgia utilizing teledermoscopy and distance learning: An ongoing report. JAAD Int. (2023) 11:140–2. doi: 10.1016/j.jdin.2023.02.010 PMC10148147 37128270

[74] Parrott R Steiner C Goldenhar L. Georgia's harvesting healthy habits: a formative evaluation. J Rural Health. (1996) 12:291–300. doi: 10.1111/j.1748-0361.1996.tb00818.x 10162860

